# Virulence genes distributed among *Staphylococcus aureus* causing wound infections and their correlation to antibiotic resistance

**DOI:** 10.1186/s12879-022-07624-8

**Published:** 2022-07-28

**Authors:** Asia Helmi Rasmi, Eman Farouk Ahmed, Abdou Mohammed Abdullah Darwish, Gamal Fadl Mahmoud Gad

**Affiliations:** 1 Microbiology and Immunology Department, Faculty of Pharmacy, Deraya University, Minia, Egypt; 2 Microbiology and Immunology Department, Faculty of Pharmacy, Sohag University, Sohag, Egypt; 3 Plastic and Reconstructive Surgery Department, Faculty of Medicine, Minia University, Minia, Egypt; 4 Microbiology and Immunology Department, Faculty of Pharmacy, Minia University, Minia, Egypt

**Keywords:** *S. aureus*, Wound infections, Virulence genes, Antibiotic resistance

## Abstract

**Background:**

*Staphylococcus aureus* causes many human infections, including wound infections, and its pathogenicity is mainly influenced by several virulence factors.

**Aim:**

This study aimed to detect virulence genes (*hla*, *sea*, *icaA*, and *fnbA*) in *S. aureus* isolated from different wound infections among Egyptian patients admitted to Minia University Hospital. This study also aimed to investigate the prevalence of these genes in methicillin-resistant *S. aureus* (MRSA), methicillin-susceptible *S. aureus* (MSSA), and vancomycin-resistant *S. aureus* isolates and the resistance and sensitivity to different antibiotic classes.

**Methods:**

A cross-sectional study was carried out from November 2019 to September 2021. Standard biochemical and microbiological tests revealed 59 *S. aureus* isolates. The Kirby-Bauer disc diffusion method was used to determine antibiotic susceptibility. DNA was extracted using a DNA extraction kit, and polymerase chain reaction was used to amplify all genes.

**Results:**

A total of 59 *S. aureus* isolates were detected from 51 wound samples. MRSA isolates accounted for 91.5%, whereas MSSA isolates accounted for 8.5%. The multidrug resistance (MDR) percentage in *S. aureus* isolates was 54.2%. *S. aureus* showed high sensitivity pattern against vancomycin, linezolid, and chloramphenicol. However, a high resistance pattern was observed against oxacillin and piperacillin. *sea* was the most predominant gene (72.9%), followed by *icaA* (49.2%), *hla* (37.3%), and *fnbA* (13.6%). *sea* was the commonest virulence gene among MRSA isolates (72.2%), and a significant difference in the distribution of *icaA* was found. However, *sea* and *icaA* were the commonest genes among MSSA isolates (79.9%). The highest distribution of *sea* was found among ciprofloxacin-resistant isolates (95.2%).

**Conclusion:**

The incidence of infections caused by MDR *S. aureus* significantly increased with MRSA prevalence. *sea* is the most predominant virulence factor among antibiotic-resistant strains with a significant correlation to piperacillin, gentamicin, and levofloxacin.

**Supplementary Information:**

The online version contains supplementary material available at 10.1186/s12879-022-07624-8.

## Introduction

The fundamental goal of the skin is to keep microbial populations on its surface under control and prevent diseases from colonizing the underlying tissue [1]. A wound is a disruption in the skin’s protective action [2]. *Staphylococcus aureus* is the most frequent opportunistic bacteria, causing many superficial and life-threatening infections [3]. It can cause various disorders, including skin and soft tissue infections (SSTIs), invasive infections, and toxin-mediated disorders [4]. Since it produces several virulence factors and acquires multidrug resistance (MDR) to various antibacterial agents, it is a major infectious agent in communities and hospitals [5].

*S. aureus* has an incredible ability to develop resistance rapidly. Environmental factors and cell membrane disruption or DNA damage can influence the fast development of antibiotic resistance [6]. More than 90% of *S. aureus* is resistant to penicillin, which remains a global issue [7]. Methicillin-resistant *S. aureus* (MRSA) is a common inhabitant of a large part of the healthy population and can cause a wide range of illnesses, from minor skin infections to life-threatening diseases [8] The MDR is defined as acquired non-susceptibility to at least one agent in three or more antimicrobial categories [9, 10]. The MDR of *S. aureus* strains has been linked to longer hospital stays, higher mortality rates, and concomitant costs [11].

The presence of many virulence factors, such as surface proteins, biofilms, exoenzymes, exotoxins, and exfoliative toxins, is linked to the ability of *S. aureus* to cause different infections. All these factors allow bacteria to attach to tissues, causing pathogenesis, and to penetrate the immune system, causing toxicity [12]. One of the virulence factors of *S. aureus* is a cytolytic, pore-forming toxin, such as α-hemolysin, which is involved in the pathogenesis of *S. aureus* [13]. Many *S. aureus* strains, particularly MRSA, release one or more distinct staphylococcal exotoxins, including staphylococcal enterotoxins [14], the most important pathogenic components belonging to the superantigen family [15].

The ability of the microorganism to successfully persist within the hospital and community and several cell wall-associated adhesive molecules, such as *fnb* (encoding fibronectin-binding protein) is responsible for the possibility of severe animal and human diseases [16, 17]. The ability of *S. aureus* to build biofilms is linked to the antimicrobial resistance mechanism. Invasion isolates are more likely to form biofilm than healthy individual carriage isolates [18] The polysaccharide intercellular adhesin (PIA) is the most important component of biofilm [19, 20]. The N-acetylglucosamyl transferase enzyme responsible for PIA synthesis is known to be encoded by *icaA* [21].

*S. aureus* persists and spreads by acquiring antibiotic resistance genes. Identification of *S. aureus* virulence genes is important for evaluation of disease development. This study focused on *S. aureus* virulence genes and to detect their correlation to antimicrobial resistance patterns.

## Materials and methods

### Study area, design, and population

A cross-sectional study was carried out from November 2019 to September 2021 in Minia University Hospital (Minia, Egypt). Wound samples were collected from the Department of Plastic and Reconstructive Surgery. The samples were properly labeled, indicating the source, sex, and age of the patient. Ethical clearance for the study was granted by Minia University Hospital.

### Collection of wound pus samples

Bacterial samples were collected from patients having wound infections present on admission to the outpatient clinic and cultured onto nutrient agar, mannitol salt agar, and DNase agar. All media were produced by Oxoid (England) and prepared according to the manufacturer’s instructions. The cultures were incubated at 37 °C for 24 h to be examined the next day.

### Isolation and identification of wound bacterial isolates

**Fig. 1:**
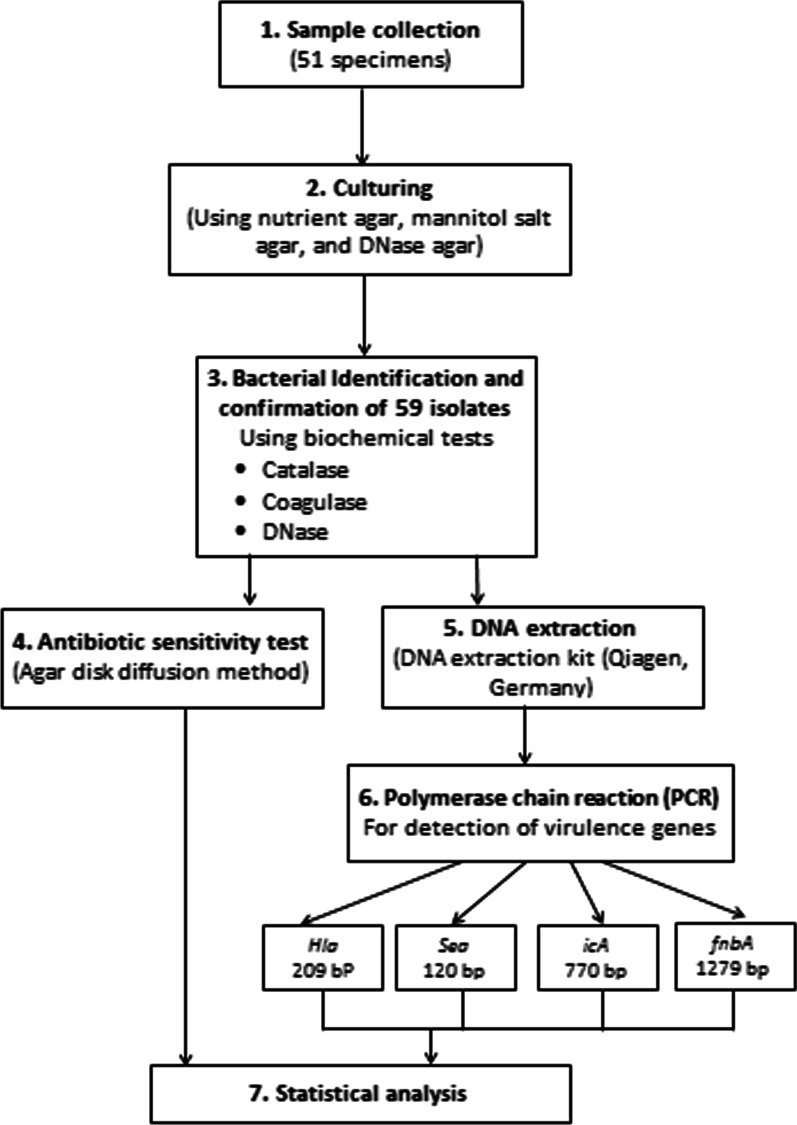
Flow chart of study procedures

The primary identification of bacterial isolates was based on colonial appearance, pigmentation, morphology, Gram staining, and biochemical characteristics. The biochemical tests applied were the standard catalase test, coagulase (tube and slide) test, and DNase test (Fig. 1). For the extended storage of bacterial isolates, preservation in 20% glycerol vials at − 70 °C was carried out.

### Antibiotic sensitivity testing

Antimicrobial sensitivity was determined by the Kirby-Bauer agar disc diffusion method according to the Clinical Laboratory Standard Institute (CLSI; 2018). Antibiotic discs were used with the following drug concentrations: linezolid (30 μg), tetracycline (30 μg), chloramphenicol (30 μg), rifampin (5 μg), piperacillin (100 μg), amoxicillin/clavulanic acid (30 μg), ampicillin/sulbactam (20 μg), levofloxacin (5 μg), gentamycin (10 μg), vancomycin (30 μg), oxacillin (1 μg), and ciprofloxacin (5 μg) were applied onto Müller-Hinton agar (Himedia). The plates were aerobically incubated at 37 °C for 24 h, and the diameter of the inhibition zones was measured (in mm). The results were compared to that of the CLSI.

### DNA extraction and detection of virulence genes

DNA was extracted using a DNA extraction kit (Qiagen, Germany), and the procedures were carried out according to the manufacturer’s instructions. The oligonucleotide primers used in this study were for the detection of genes encoding α-hemolysin (*hla*), staphylococcal enterotoxin A (*sea*), intracellular adhesion A (*icaA*), and fibronectin-binding protein A (*FnbA*). Table 1 lists the primer sequences (Metabione, Germany) of this study, and Table 2 presents the conditions of the polymerase chain reaction (PCR) products. The PCR products were resolved by electrophoresis on 1% agarose gel, and electrophoresis was carried out at a constant current of 50 mA for 30 min. DNA bands were visualized by ethidium bromide staining and ultraviolet transillumination light. The size of the fragments was determined by comparing their migration to a 100 bp ladder as a standard.

**Table 1:** The list of primers sequences

Virulence genes	Primer Sequence	References
Hemolysin A (*hla*)	F: CTG ATT ACT ATC CAA GAA ATT CGA TTG R: CTT TCC AGC CTA CTT TTT TAT CAG T	[48]
Staphylococcal enterotoxin A (*sea*)	F: TTG GAA ACG GTT AAA ACG AA R: GAA CCT TCC CAT CAA AAA CA	[49]
Intracellular adhesion-A (*icaA*)	F: GAT TAT GTA A TG TGC TTG GA R:ACT ACT GCT GCG TTA ATA AT	[50]
Fibronectin binding protein-A (*fnbA*)	F: GCG GAG ATC AAA GAC AA R: CCA TCT ATA GCT GTG TGG	[51]

**Table 2:** Conditions of PCR products

Gene	Initial Denaturation	Denaturation	Annealing	Extension	Final Extension	Cycles	Product size (bp)
*hla*	95 °C for 5 min	95 °C for 50 s	58 °C for 30 s	72 °C for 1 min	72 °C for 10 min	40	209 bp
*sea*	95 °C for 5 min	95 °C for 1 min	55 °C for 45 s	72 °C for 1 min	72 °C for 10 min	40	120 bp
*icaA*	95 °C for 5 min	95 °C for 1 min	50 °C for 1 min	72 °C for 1.5 min	72 °C for 5 min	40	770 bp
*fnbA*	95 °C for 5 min	95 °C for 1 min	47 °C for 1 min	72 °C for 1.5 min	72 °C for 5 min	40	1279 bp

### Statistical analysis

Statistical analyses were performed using χ^2^ using SPSS version 16 (SPSS, Inc., Chicago, IL, USA). A χ^2^ test was used to test the association between *S. aureus* virulence genes and participant's gender and age as well as with the antibiotic resistance profile. Similarly, the association between the antibiotic resistance profile with participant's gender and age groups was detected. The results were considered statistically significant when *P* ≤ 0.05.

## Results

### Prevalence of *S. aureus* isolates according to gender, age, and sample source

**Fig. 2:**
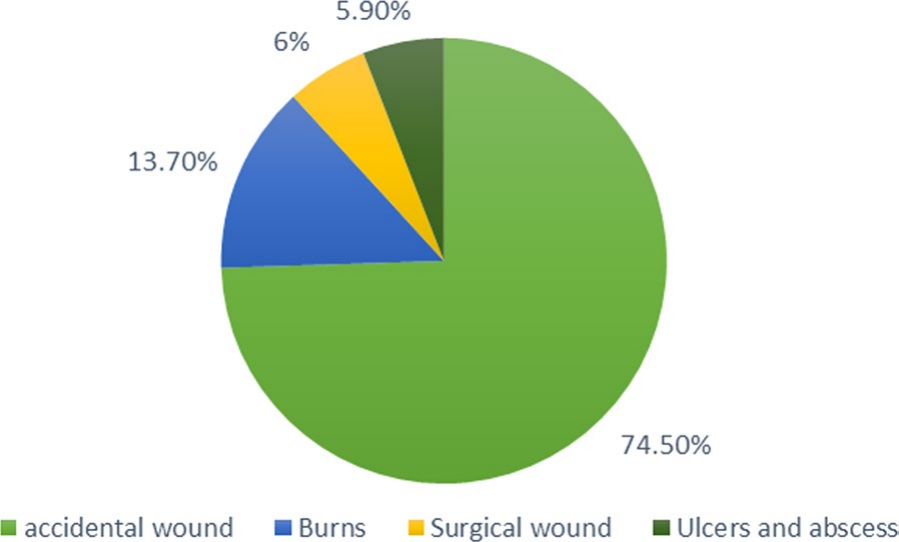
Prevalence of *S. aureus* isolated from patients from different types of wound infections

A total of 59 *S. aureus* isolates were detected from 51 different wound samples. The incidence of *S. aureus* was much higher in males [n = 36 (70.6%)] than in females [n = 15 (29.4%)]. Patients were classified into different age groups from 1 month to 60 years (mean ± standard deviation, 28.98 ± 16.95). The highest prevalence of *S. aureus* was observed in the age group between 1 and 20 years (45.1%), followed by patients in the age group between 41 and 60 years (29.4%) and finally patients in the age group from 21 to 40 years (25.5%). Figure 2 shows that the highest number of samples was from accidental wounds, such as animal bites, occupational injuries, a sharp tool, or car accidents (n = 38; 74.5%), followed by seven samples of burn infection (burning agents, such as flame, scald, electrical, boiled water, and chemical reagent; 13.7%). Three samples were from surgical wounds (5.9%) and three samples were from ulcers and abscess discharge (5.9%).

### Antimicrobial sensitivity testing

Table 3 shows that MRSA isolates accounted for 91.5%, whereas methicillin-susceptible *S. aureus* (MSSA) isolates accounted for 8.5%. *S. aureus* had low resistance to chloramphenicol (10.2%), vancomycin (13.5%), and linezolid (16.9%). Moderate (intermediate) resistance was recorded against gentamycin (33.9%), levofloxacin and ciprofloxacin (both 35.6%), rifampin (37.3%), and tetracycline (62.7%). High resistance was observed against oxacillin, amoxicillin/clavulanic acid, ampicillin/sulbactam (all 91.5%), and piperacillin (100%). The MDR in *S. aureus* isolates was 54.2%. *S. aureus* had R0 = 0%, R1 = 20.3%, R2 = 23.7%, R3 = 16.9%, R4 = 13.6%, R5 = 16.9%, R6 = 1.7%, R7 = 3.4%, and R8 = 1.7% (R0 represents the number of isolates sensitive to all antimicrobial classes tested, whereas R = 1, 2, 3, 4, 5, 6, 7, and 8 represent isolates resistant to 1, 2, 3, 4, 5, 6, 7, and 8 antibiotic classes, respectively). No statistically significant difference was detected between the resistance profile of tested antibiotics and participant’s gender or age group (p > 0.05) (Tables 4 and 5).

**Table 3:** Antibiotic sensitivity profile of *S. aureus* isolates

Antibiotic	Concentration μg /disc	Sensitive No. (%)	Intermediate No. (%)	Resistant No. (%)	Company
Linezolid	30 μg	49 (83.1%)	0 (0%)	10 (16.9%)	Bioanalyse limited -Turkey
Tetracycline	30 μg	16 (27.1%)	6 (10.2%)	37 (62.7%)	Himedia, India
Chloramphenicol	30 μg	42 (71.2%)	11 (18.6%)	6 (10.2%)	Bioanalyse limited -Turkey
Rifampin	5 μg	34 (57.6%)	3 (5.1%)	22 (37.3%)	Himedia, India
Piperacillin	100 μg	0 (0%)	0 (0%)	59 (100%)	Bioanalyse limited -Turkey
Gentamycin	10 μg	29 (49.2%)	10 (16.9%)	20 (33.9%)	Himedia, India
Ampicillin / Sulbactam	20 μg (10/10 μg)	5 (8.5%)	0 (0%)	54 (91.5%)	Bioanalyse limited -Turkey
Oxacillin	1 μg	5 (8.5%)	0 (0%)	54 (91.5%)	Sigma, USA
Levofloxacin	5 μg	33(55.9%)	5 (8.5%)	21 (35.6%)	Himedia, India
Ciprofloxacin	5 μg	31 (52.2%)	7 (11.9%)	21 (35.6%)	Bioanalyse limited -Turkey
Amoxicillin/ Clavulanic	30 μg (20/10 μg)	5 (8.5%)	0 (0%)	54 (91.5%)	Bioanalyse limited -Turkey
Vancomycin	30 μg	51 (86.4%)	0 (0%)	8 (13.5%)	Sigma, USA

**Table 4:** Correlation between antibiotic resistance profile and patients’ gender

Antibiotic	Resistance profile			p*
	Sensitive	Intermediate	Resistant	
*Linezolid*				
Male	5	31	0	0.111
Female	5	10	0	
*Tetracycline*				
Male	25	7	4	0.888
Female	11	3	1	
*Chloramphenicol*				
Male	6	25	5	0.626
Female	1	12	2	
*Rifampin*				
Male	14	19	2	0.995
Female	6	8	1	
*Piperacillin*				
Male	36	0	0	N/A
Female	15	0	0	
*Gentamycin*				
Male	15	17	4	0.321
Female	4	7	4	
*Amoxicillin/Sulbactam*				
Male	34	2	0	0.878
Female	14	1	0	
*Oxacillin*				
Male	34	2	0	0.878
Female	14	1	0	
*Levofloxacin*				
Male	16	19	1	0.579
Female	5	10	0	
*Ciprofloxacin*				
Male	15	18	3	0.780
Female	15	8	2	
*Amoxicillin/Clavulanic acid*				
Male	34	2	0	0.878
Female	14	1	0	
*Vancomycin*				
Male	5	31	0	0.585
Female	3	12	0	

*Chi-square test; P-value was set to 0.05; N/A: not applicable

**Table 5:** Correlation between antibiotic resistance profile and patients age groups

Antibiotic	Resistance profile			p*
	Sensitive	Intermediate	Resistant	
*Linezolid*				
1 to 20	4	19	0	0.595
21 to 40	4	12	0	
41 to 60	4	10	0	
*Tetracycline*				
1 to 20	18	4	1	0.617
21 to 40	8	4	2	
41 to 60	10	2	2	
*Chloramphenicol*				
1 to 20	2	19	2	0.618
21 to 40	3	9	2	
41 to 60	2	9	3	
*Rifampin*				
1 to 20	12	9	1	0.395
21 to 40	3	10	1	
41 to 60	5	8	1	
*Piperacillin*				
1 to 20	23	0	0	N/A
21 to 40	14	0	0	
41 to 60	14	0	0	
*Gentamycin*				
1 to 20	10	10	3	0.851
21 to 40	4	8	2	
41 to 60	5	6	3	
*Amoxicillin/Sulbactam*				
1 to 20	22	1	0	0.915
21 to 40	13	1	0	
41 to 60	13	1	0	
*Oxacillin*				
1 to 20	22	1	0	0.915
21 to 40	13	1	0	
41 to 60	13	1	0	
*Levofloxacin*				
1 to 20	11	12	0	0.450
21 to 40	4	9	1	
41 to 60	0	8	0	
*Ciprofloxacin*				
1 to 20	11	11	1	0.439
21 to 40	5	8	1	
41 to 60	4	7	3	
*Amoxicillin/Clavulanic acid*				
1 to 20	22	1	0	0.915
21 to 40	13	1	0	
41 to 60	13	1	0	
*Vancomycin*				
1 to 20	4	19	0	0.556
21 to 40	1	13	0	
41 to 60	3	11	0	

*Chi-square test; P-value was set to 0.05; N/A: not applicable

### Detection of virulence genes

**Fig. 3:**
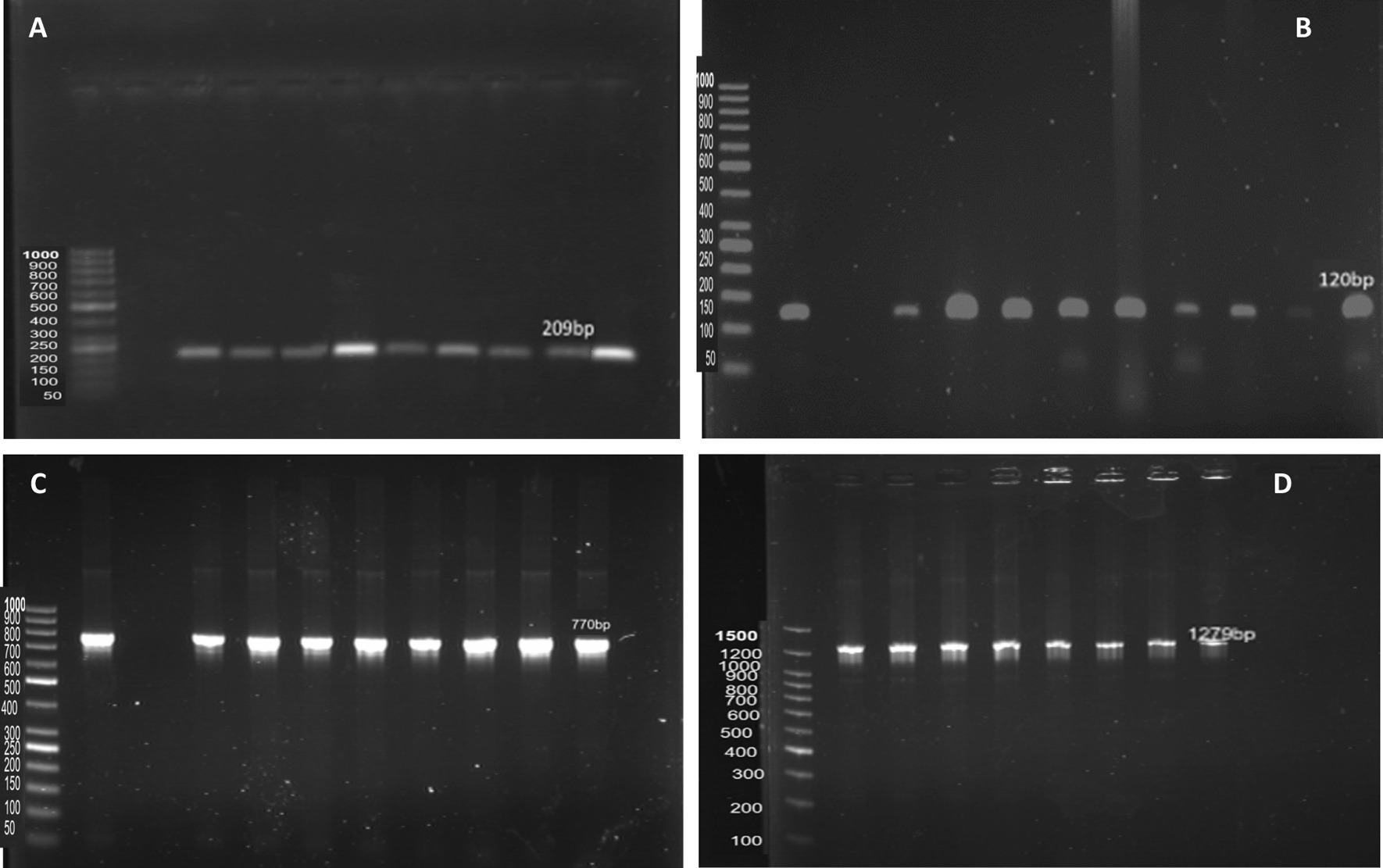
Detection of amplification product of different virulence genes: **A** *hla* gene by PCR; lane 1: negative control, lane 2: positive control and lanes 3 to 10: positive PCR products (209 bp); **B** *sea* gene by PCR; lane 1: positive control, lane 2: negative control and lanes 3 to 11: positive PCR products (120 bp); **C** *icaA* gene by PCR; lane 1: positive control, lane 2: negative control and lanes 3 to 10: positive PCR products (770 bp); **D** *fnbA* gene by PCR; lanes 1 to 7: positive PCR products (1279 bp), lane 8: positive control and lane 9: negative control

To test the virulence genes of the isolates in this study, *hla*, *sea*, *icaA*, *and fnbA* were detected by PCR amplification. Table 6 shows that *sea* was the most predominant in 72.9% of the isolates. *icaA* was found in 49.2% of the isolates, followed by *hla* (37.3% of the isolates) and *fnbA* (13.6% of the isolates). Amplicon sizes of 209, 120, 770, and 1279 bp were considered positive for the presence of *hla*, *sea*, *icaA*, and *fnbA*, respectively. Figure 3 shows *hla*, *sea*, *icaA*, and *fnbA* PCR amplification products among *S. aureus* isolates, respectively. *sea* was the commonest virulence gene among MRSA and vancomycin-resistant *S. aureus* (VRSA) isolates (72.2% and 62.5%, respectively). However, *sea* and *icaA* were the commonest genes among MSSA isolates (80%; Table 6).

**Table 6:** Frequencies of virulence genes among MRSA, MSSA and VRSA strains

Virulence genes	Total No. = 59 (%)	MRSA No. = 54 (%)	MSSA No. = 5 (%)	VRSA No. = 8 (%)
*hla*	22 (7.3)	20 (36.9)	2 (39.9)	3 (37.5)
*sea*	43 (72.9)	39 (72.2)	4 (79.9)	5 (62.5)
*icaA*	29 (49.2)	25 (46.3)	4 (79.9)	4 (49.9)
*fnbA*	8 (13.6)	6 (11.1)	2 (39.9)	0 (0)

A significant correlation was observed between virulence genes (*hla*, *sea* and *icaA*) and patients age groups (*P* < 0.05). While no statistically significant difference was detected between the tested virulence genes and participant’s gender (Table 7).

**Table 7:** Correlation between virulence genes and patients gender and age groups

	*hla*		P*	*sea*		P*	*fnb*		P*	*icaA*		P*
	** + **	**−**		** + **	**−**		** + **	**−**		** + **	**−**	
*Gender*												
Male	16	20	0.770	29	7	0.125	6	30	0.766	20	16	0.311
Female	6	9		9	6		2	13		6	9	
*Age group*												
1 to 20	10	1	**0.002**	20	3	**0.006**	5	18	0.489	16	7	**0.041**
21 to 40	6	8		12	2		2	12		6	8	
41 to 60	6	8		6	8		1	13		4	10	

The bold values indicate a statisticatly significant difference. The alpha level of siginificance was set to 0.05

*Chi-square test; P-value was set to 0.05

Gene present ( +); gene absent (−)

*sea* was the commonest virulence gene among antibiotic-resistant and antibiotic-sensitive isolates, followed by *icaA*, *hla*, and *fnbA*. The highest distribution of *sea* was among the ciprofloxacin (95.2%)-, gentamycin (89.9%)-, and tetracycline (75.7%)-resistant isolates. At the same time, the highest distribution of *sea* was among oxacillin (79.9%)-, linezolid (75.5%)-, and rifampin (73.5%)-sensitive isolates (Table 8).

**Table 8:** Correlation between *S. aureus* virulence genes and antibiotic resistance

Antibiotic	*hla*			*sea*			*icaA*			*fnbA*		
	R	S	p*	R	S	p*	R	S	p*	R	S	p*
*Linzolid*												
Positive gene	4 (39.9%)	18 (36.7%)	0.846	5 (50%)	37 (75.5%)	0.105	4 (39.9%)	25 (51%)	0.525	0 (0%)	8 (16.3%)	0.169
Negative gene	6 (59.9%)	31 (63.3%)		5 (50%)	12 (24.5%)		6 (59.9%)	24 (48.9%)		10 (100%)	41 (83.7%)	
*Tetracycline*												
Positive gene	14 (37.8%)	5 (31.3%)	0.646	28 (75.7%)	11 (68.8%)	0.6	21 (56.8%)	6 (37.5%)	0.198	4 (10.8%)	2 (12.5)	0.602
Negative gene	23 (62.2%)	11 (68.8%)		9 (24.3%)	5 (31.3%)		16 (43.2%)	10 (62.5%)		33 (89.2%)	14 (87.5%)	
*Chloramphenicol*												
Positive gene	2 (33.3%)	14 (33.3%)	1	4 (66.7%)	30 (71.4%)	0.81	3 (49.9%)	20 (47.6%)	0.913	0 (0%)	5 (12%)	0.372
Negative gene	4 (66.7%)	28 (66.7%)		2 (33.3%)	12 (28.6%)		3 (49.9%)	22 (52.4%)		6 (100%)	37 (88.1%)	
*Rifampin*												
Positive gene	6 (27.3%)	15 (44.1%)	0.203	16 (72.7%)	25 (73.5%)	0.947	11 (49.9%)	17 (49.9%)	1	3 (13.6%)	5 (14.7%)	0.911
Negative gene	16 (72.7%)	19 (55.9%)		6 (27.3%)	9 (26.5%)		11 (49.9%)	17 (49.9%)		19 (86.4%)	29 (85.3%)	
*Piperacillin*												
Positive gene	22 (37.3%)	0 (0%)	** < 0.0001**	43 (72.9%)	0 (0%)	** < 0.0001**	29 (49.2%)	0 (0%)	** < 0.0001**	8 (13.6%)	0 (0%)	** < 0.0001**
Negative gene	37 (62.7%)	0 (0%)		16 (27.1%)	0 (0%)		30 (50.4%)	0 (0%)		51 (86.4%)	0 (0%)	
*Gentamycin*												
Positive gene	3 (14.9%)	15 (51.7%)	**0.005**	18 (89.9%)	18 (62.1%)	**0.03**	12 (59.9%)	13 (44.8%)	0.296	3 (14.9%)	5 (17.2%)	0.835
Negative gene	17 (84.9%)	14 (48.3%)		2 (9.9%)	11 (37.9%)		8 (39.9%)	16 (55.2%)		17 (84.9%)	17 (58.6%)	
*Ampicillin / Sulbactam*												
Positive gene	20 (36.9%)	2 (39.9%)	0.896	39 (72.2%)	4 (79.9%)	0.708	25 (46.3%)	4 (79.9%)	**0.044**	6 (11.1%)	2 (39.9%)	0.071
Negative gene	34 (62.9%)	3 (59.9%)		15 (27.8%)	1 (19.9%)		19 (35.2%)	1 (19.9%)		48 (88.9%)	3 (59.9%)	
*Oxacillin*												
Positive gene	20 (36.9%)	2 (39.9%)	0.896	39 (72.2%)	4 (79.9%)	0.708	25 (46.3%)	4 (79.9%)	**0.044**	6 (11.1%)	2 (39.9%)	0.071
Negative gene	34 (62.9%)	3 (59.9%)		15 (27.8%)	1 (19.9%)		19 (35.2%)	1 (19.9%)		48 (88.9%)	3 (59.9%)	
*Levofloxacin*												
Positive gene	4 (18.9%)	17 (51.5%)	**0.017**	19 (90.5%)	21 (63.6%)	**0.028**	10 (4.8%)	16 (48.5%)	0.951	2 (9.5%)	5 (15.2%)	0.548
Negative gene	17 (80.9%)	16 (48.5%)		2 (9.5%)	12 (36.4%)		11 (52.3%)	17 (51.5%)		19 (90.5%)	28 (84.9%)	
*Ciprofloxacin*												
Positive gene	5 (23.8%)	13 (41.9%)	0.178	20 (95.2%)	20 (64.5%)	**0.01**	11 (52.4%)	14 (45.2%)	0.609	4 (18.9%)	4 (12.9%)	** < 0.0001**
Negative gene	16 (76.2%)	18 (58.1%)		1 (4.8%)	11 (35.5%)		10 (47.6%)	17 (54.8%)		17 (80.9%)	27 (87.1%)	
*Amoxicillin/ Clavulanic*												
Positive gene	20 (36.9%)	2 (39.9%)	0.896	39 (72.2%)	4 (79.9%)	0.708	25 (46.3%)	4 (79.9%)	**0.044**	6 (11.1%)	2 (39.9%)	0.071
Negative gene	34 (62.9%)	3 (59.9%)		15 (27.8%)	1 (19.9%)		19 (35.2%)	1 (19.9%)		48 (88.9%)	3 (59.9%)	
*Vancomycin*												
Positive gene	3 (37.5%)	19 (37.3%)	0.989	5 (62.5%)	37 (72.5%)	0.56	4 (49.9%)	25 (48.9%)	0.959	0 (0%)	8 (15.7%)	0.228
Negative gene	5 (62.5%)	32 (62.7%)		3 (37.5%)	14 (27.5%)		4 (49.9%)	26 (50.9%)		8 (100%)	43 84.3%)	

The bold values indicate a statisticatly significant difference. The alpha level of siginificance was set to 0.05

^*^Chi-square test

P-values was set to 0.05

A statistically significant correlation (*P* < 0.05) was detected between the presence and absence of *hla* and *sea* and piperacillin, gentamicin, and levofloxacin resistance and sensitivity. However, a significant difference in the distribution of *icaA* was found among β-lactam-resistant and β-lactam-sensitive isolates. *fnbA* was significantly associated with piperacillin and ciprofloxacin resistance and sensitivity (Table 8). Table 9 shows that *sea* and *icaA* had the highest coexistence (40.7%), followed by *sea* and *hla* (21.9%).

**Table 9:** Coexistence of virulence genes among *S. aureus* isolates

Virulence genes	Distribution No. (%)
*hla* + *sea*	13 (21.9%)
*hla* + *sea* + *fnbA*	4 (6.8%)
*sea* + *fnbA*	8 (13.6%)
*hla* + *fnbA*	4 (6.8%)
*icaA* + *hla*	11 (18.6%)
*icaA* + *sea*	24 (40.7%)
*icaA* + *fnbA*	5 (8.5%)
*hla* + *sea* + *icaA* + *fnbA*	3 (5.1%)

## Discussion

*S. aureus* is the commonest pathogenic bacteria found in different wound specimens [22, 23]. Muluye et al. [24] stated that the prevalence of *S. aureus* in males and females was 38.1% and 28.7%, respectively. The first result was much lower than in this study, whereas the second was similar to this study. Patients were classified into different age groups from 1 month to 60 years. The highest prevalence of *S. aureus* (45.1%) and examined virulence genes were observed in the age group between 1 and 20 years. In the same time, there is a significant association between tested virulence genes and patients age groups. Torpy et al. [25] stated that the high prevalence of *S. aureus* in the age group between 1 and 20 years was because most young males (< 20 years) in the country have traditionally worked in occupations such as agriculture, construction, transportation, and industries, all of which are likely to expose them to trauma and different wound infections.

In this study, the highest prevalence of *S. aureus* was found in trauma and accidental wound infections, similar to other studies [26, 27], suggesting that the rate of *S. aureus* isolates in open wound infection was 76.9%, similar to these findings.

The predominant isolate *S. aureus* was sensitive to vancomycin (100%) [28], supporting the findings that considered vancomycin as one of the drugs with high susceptibility pattern against *S. aureus*. The same study revealed that *S. aureus* showed a high level of resistance to penicillin and oxacillin (84.6% and 76.9%, respectively). Although these results were lower, they supported this study. They considered piperacillin and oxacillin as drugs with high resistance patterns against *S. aureus* along with ampicillin/sulbactam and amoxicillin/clavulanic acid.

Linezolid is an efficient antibiotic for treating *S. aureus* infections among four burn centers [23], in agreement with this study*.* The sensitivity rate of chloramphenicol against *S. aureus* was 71.2%, similar to another study [29] that showed a 68.4% sensitivity rate for chloramphenicol. The notable sensitivity of *S. aureus* to vancomycin, linezolid, and chloramphenicol could be linked to a lower use of these antibiotics due to their shortage availability in the market, high costs, and toxic side effects [30].

Many studies showed a high MRSA prevalence in wound infections [31] and reported a high rate of MRSA and VRSA (44.6% and 61.5%, respectively). The finding for MRSA was lower than in this study, whereas the findings for VRSA were much higher. Moreover, the MRSA results in this study disagreed with Bessa et al. [22], who suggested that 21.8% of *S. aureus* was resistant to oxacillin. This study also revealed a remarkable increase in MRSA compared to a previous study by Ahmed et al. [32], who reported a 24% MRSA prevalence in the same hospital 10 years ago. This raised the alarm about the escalating and noticeable increase in MRSA prevalence in Egypt. The increase of MRSA in wound infections has contributed to high treatment costs and longer hospital stays, which have major implications for infection management, particularly in developing countries. These findings contribute to a worrying situation in the Minia Government regarding MRSA expansion. The necessity for more detailed molecular epidemiologic surveillance studies on MRSA and VRSA in the next years is critical.

The MDR of *S. aureus* isolates was 54.2%, similar to other studies [33, 34], which reported 54.9% and 47.9%, respectively. However, another study [28] stated that *S. aureus* showed 94.8%, higher than this study. Low activity of commonly used antibiotics, such as amoxicillin/clavulanic acid, ampicillin/sulbactam, oxacillin, and piperacillin, may be due to increased consumption of a particular class of antibiotics, resulting in resistance due to mutation(s) at drug target sites or the disruption of drug accumulation in the cytoplasm caused by cell wall rearrangement [31–36]. As a result, they are no longer effective in treating wound infections.

The incidence of some major virulence indicators of *S. aureus* in wound specimens was examined in this study. This study concentrated on a small number of genes linked to *S. aureus* pathogenicity. These genes (*hla*, *sea*, *icaA*, and *fnbA*) were chosen because they were the most frequent in aggressive isolates. These targeted genes spread across the isolates after PCR amplification. Furthermore, the bulk of the isolates demonstrated a wide range of gene combinations, indicating that the study sample has a level of genetic diversity.

Antimicrobial resistance and virulence factor genes showed significant relationships in this study. This finding could be explained by the proximity location of the resistance gene to the virulence gene [31, 37].

The predominant virulence and inducible resistance genes in MRSA and MSSA isolates were related to *sea* [38, 39]. All previous studies supported this study because *sea* is the commonest among MRSA and MSSA isolates. Cavalcante et al. [40] reported that the prevalence of *sea* in *S. aureus* isolates collected from infected skin lesions of atopic dermatitis children was 76.4% in total *S. aureus* isolates, 73.9% in MRSA isolates, and 78.1% in MSSA isolates, in agreement with this study. Li et al. [41] reported that the frequency of *sea* in *S. aureus* isolates from SSTIs in children was 0%, which was totally opposite to this study.

PCR investigation revealed that *hla* was found in 30.5% of 85 *S. aureus* isolated from various clinical sources [42], close to the present findings. The prevalence of *icaA* in MRSA was 60.3% [43], which was slightly higher than the present data. The prevalence of *fnbA* was 4.9% and 19.9% in MRSA and MSSA strains, respectively [44]. The prevalence of *fnbA* in MRSA was close to this study, whereas *fnbA* in MSSA was much lower than in the present data. Another study [45] suggested that the incidence of *fnbA* in wound swabs was 28.8%, which was slightly higher than the present results. The prevalence of *fnbA* and *icaA* in burn units was 2.9% and 44.9%, respectively [46]. *fnbA* was slightly lower than in this study, whereas the percentage of *icaA* was similar to the present data. The incidence of *fnbA* in MRSA and MSSA strains was 15.5% and 36.9%, respectively. However, the incidence of *icaA* in MRSA and MSSA was 84.5% and 78.3%, respectively [38]. The percentages of *fnbA* were similar to this study. However, the percentage of *icaA* was much higher in MRSA but was similar to the present data in MSSA. The prevalence of *sea* was 11.8% in amoxicillin/clavulanic acid and oxacillin susceptibility samples, 9.2% in rifampin, 0% in penicillin, 88.2% in chloramphenicol, and 100% in vancomycin [47]. The first three percentages were much lower than in this study. However, the percentages of chloramphenicol and vancomycin were slightly higher than in this study. The percentage of the coexistence of *sea* and *hla* was 36.9% [17], which was slightly higher than in this study.

The limitation of this study was the inability to detect more virulence genes and express the chosen virulence factors by molecular typing of the isolates (Additional file 1, Additional file 2).

## Conclusions

Within the limitations of the current study, it can be concluded that the challenging, increasingly difficult, and widespread bacterial resistance to antibiotics has developed, the incidence of infections caused by MDR *S. aureus* has increased. The prevalence of CA-MRSA was high among patients with various wound infections. Bacterial resistance profile was the least against vancomycin and linezolid effective antibiotics. The correlation between CA-MRSA strain virulence genes distribution and antibiotic resistance profile showed high incidence of *sea* and *icaA* genes. All virulence genes were significantly distributed among piperacillin resistant isolates. β-lactam resistant isolates showed a significant correlation with *IcaA virulence* gene. After the emergence of high percentage of *sea* among ciprofloxacin resistant isolates, we expect that more genes will appear in future studies regarding *S. aureus* virulence genes. Therefore, the spread of bacterial resistance must be monitored in hospitals by using antibacterial agents properly to avoid more complications and to keep the empirical medications as effective as they are.

## Supplementary Information

**Additional file 1: Figure S1.** Detection of amplification product of *hla* gene by PCR; lane 1: negative control, lane 2: positive control and lanes 3 to 10: positive PCR products (209bp). **Figure S2.** Detection of amplification product of *sea* gene by PCR; lane 1: positive control, lane 2: negative control and lanes 3 to 11: positive PCR products (120bp). **Figure S3.** Detection of amplification product of *icaA* gene by PCR; lane 1: positive control, lane 2: negative control and lanes 3 to 10: positive PCR products (770bp). **Figure S4.** Detection of amplification product of *fnbA* gene by PCR; lanes 1 to 7: positive PCR products (1279bp), lane 8: positive control and lane 9: negative control

**Additional file 2: Table S1.** Biochemical tests results of wound infection isolates.

## Data Availability

The data sets generated and/or analyzed during this study are not publicly available due to privacy but are available from the corresponding author (A.E.F.) on reasonable request.

## References

[1] Ndip RN, Takang A, Echakachi CM, Malongue A, Akoachere J, Ndip LM, Luma HN (2007). In-vitro antimicrobial activity of selected honeys on clinical isolates of Helicobacter pylori. Afr Health Sci..

[2] Leaper D, Harding K (1998). Wounds: biology and management.

[3] Tong SY, Davis JS, Eichenberger E, Holland TL, Fowler VG (2015). Staphylococcus aureus infections: epidemiology, pathophysiology, clinical manifestations, and management. Clin Microbiol Rev.

[4] Morell EA, Balkin DM (2010). Methicillin-resistant Staphylococcus aureus: a pervasive pathogen highlights the need for new antimicrobial development. Yale J Biol Med.

[5] Gauliard E, Ouellette SP, Rueden KJ, Ladant D (2015). Characterization of interactions between inclusion membrane proteins from *Chlamydia trachomatis*. Front Cell Infect Microbiol.

[6] McCallum N, Berger-Bächi B, Senn MM (2010). Regulation of antibiotic resistance in *Staphylococcus aureus*. Int J Med Microbiol.

[7] Bagdonas R, Tamelis A, Rimdeika R (2003). Staphylococcus aureus infection in the surgery of burns. Medicina.

[8] Lewis K (2001). Riddle of biofilm resistance. Antimicrob Agents Chemother.

[9] Magiorakos AP, Srinivasan A, Carey RB, Carmeli Y, Falagas ME, Giske CG, Harbarth S, Hindler JF, Kahlmeter G, Olsson-Liljequist B (2012). Multidrug-resistant, extensively drug-resistant and pandrug-resistant bacteria: an international expert proposal for interim standard definitions for acquired resistance. Clin Microbiol Infect.

[10] Wolfensberger A, Kuster SP, Marchesi M, Zbinden R, Hombach M (2019). The effect of varying multidrug-resistence (MDR) definitions on rates of MDR gram-negative rods. Antimicrob Resist Infect Control.

[11] Rauber JM, Carneiro M, Arnhold GH, Zanotto MB, Wappler PR, Baggiotto B, Valim AR, d'Azevedo PA (2016). Multidrug-resistant *Staphylococcus* spp and its impact on patient outcome. Am J Infect Control.

[12] Costa AR, Batistão DW, Ribas RM, Sousa AM, Pereira MO, Botelho CM, Mendez-Vilas A (2013). *Staphylococcus aureus* virulence factors and disease. Microbial pathogens and strategies for combating them: science, technology and education.

[13] Labandeira-Rey M, Couzon F, Boisset S, Brown EL, Bes M, Benito Y, Barbu EM, Vazquez V, Höök M, Etienne J (2007). Staphylococcus aureus Panton-Valentine leukocidin causes necrotizing pneumonia. Science.

[14] Llewelyn M, Cohen J (2002). Superantigens: microbial agents that corrupt immunity. Lancet Infect Dis.

[15] Pinchuk IV, Beswick EJ, Reyes VE (2010). Staphylococcal enterotoxins. Toxins.

[16] Rahimi F, Katouli M, Karimi S (2016). Biofilm production among methicillin resistant *Staphylococcus aureus* strains isolated from catheterized patients with urinary tract infection. Microb Pathog.

[17] Motallebi M, Jabalameli F, Asadollahi K, Taherikalani M, Emaneini M (2016). Spreading of genes encoding enterotoxins, haemolysins, adhesin and biofilm among methicillin resistant *Staphylococcus aureus* strains with staphylococcal cassette chromosome mec type IIIA isolated from burn patients. Microb Pathog.

[18] Vandecasteele S, Peetermans W, Merckx R, Rijnders B, Van Eldere J (2003). Reliability of the ica, aap and atlE genes in the discrimination between invasive, colonizing and contaminant Staphylococcus epidermidis isolates in the diagnosis of catheter-related infections. Clin Microbiol Infect.

[19] Lappin-Scott HM, Bass C (2001). Biofilm formation: attachment, growth, and detachment of microbes from surfaces. Am J Infect Control.

[20] Ziebuhr W, Heilmann C, Götz F, Meyer P, Wilms K, Straube E, Hacker J (1997). Detection of the intercellular adhesion gene cluster (ica) and phase variation in Staphylococcus epidermidis blood culture strains and mucosal isolates. Infect Immun.

[21] Arciola CR, Baldassarri L, Montanaro L (2001). Presence of icaA and icaD genes and slime production in a collection of staphylococcal strains from catheter-associated infections. J Clin Microbiol.

[22] Bessa LJ, Fazii P, Di Giulio M, Cellini L (2015). Bacterial isolates from infected wounds and their antibiotic susceptibility pattern: some remarks about wound infection. Int Wound J.

[23] Chen X, Yang H-H, Huangfu Y-C, Wang W-K, Liu Y, Ni Y-X, Han L-Z (2012). Molecular epidemiologic analysis of *Staphylococcus aureus* isolated from four burn centers. Burns.

[24] Muluye D, Wondimeneh Y, Ferede G, Nega T, Adane K, Biadgo B, Tesfa H, Moges F (2014). Bacterial isolates and their antibiotic susceptibility patterns among patients with pus and/or wound discharge at Gondar university hospital. BMC Res Notes.

[25] Torpy JM, Burke A, Glass RM (2005). Wound infections. JAMA.

[26] Shittu A, Kolawole D, Oyedepo E (2002). A study of wound infections in two health institutions in Ile-Ife. Nigeria. Afr J Biomed Res..

[27] Kihla AJ-FT, Ngunde PJ, Mbianda SE, Nkwelang G, Ndip RN (2014). Risk factors for wound infection in health care facilities in Buea, Cameroon: aerobic bacterial pathogens and antibiogram of isolates. Pan Afr Med J.

[28] Mohammed A, Seid ME, Gebrecherkos T, Tiruneh M, Moges F (2017). Bacterial isolates and their antimicrobial susceptibility patterns of wound infections among inpatients and outpatients attending the University of Gondar Referral Hospital, Northwest Ethiopia. Int J Microbiol..

[29] Damen JG, Faruk S, Dancha C (2015). Aerobic bacteria isolates of septic wound infections and their antibiogram in North Central Nigeria..

[30] Mama M, Abdissa A, Sewunet T (2014). Antimicrobial susceptibility pattern of bacterial isolates from wound infection and their sensitivity to alternative topical agents at Jimma University Specialized Hospital, South-West Ethiopia. Ann Clin Microbiol Antimicrob.

[31] Stotts SN, Nigro OD, Fowler TF, Roger S, Steward GF. Virulence and antibiotic resistance gene combinations among staphylococcus aureus isolates from costal waters of Oahu, Hawaii. J Young Investig. 2005.

[32] Ahmed EF, Gad GF, Abdalla AM, Hasaneen AM, Abdelwahab SF (2014). Prevalence of methicillin resistant Staphylococcus aureus among Egyptian patients after surgical interventions. Surg Infect.

[33] Khadri H, Alzohairy M (2010). Prevalence and antibiotic susceptibility pattern of methicillin-resistant and coagulase-negative staphylococci in a tertiary care hospital in India. Int J Med Med Sci.

[34] Hailu D, Derbie A, Mekonnen D, Zenebe Y, Adem Y, Worku S, Biadglegne F (2016). Drug resistance patterns of bacterial isolates from infected wounds at Bahir Dar regional Health Research Laboratory center, Northwest Ethiopia. Ethiop J Health Dev.

[35] Barker KF (1999). Antibiotic resistance: a current perspective. Br J Clin Pharmacol.

[36] Speer BS, Shoemaker NB, Salyers AA (1992). Bacterial resistance to tetracycline: mechanisms, transfer, and clinical significance. Clin Microbiol Rev.

[37] Campbell TL, Henderson J, Heinrichs DE, Brown ED (2006). The yjeQ gene is required for virulence of *Staphylococcus aureus*. Infect Immun.

[38] Tabandeh M, Kaboosi H, Armaki MT, Pournajaf A, Ghadikolaii FP (2022). New update on molecular diversity of clinical Staphylococcus aureus isolates in Iran: Antimicrobial resistance, adhesion and virulence factors, biofilm formation and SCCmec typing. Mol Biol Rep..

[39] Argudín MÁ, Mendoza MC, Vazquez F, Rodicio MR (2011). Exotoxin gene backgrounds in bloodstream and wound *Staphylococcus aureus* isolates from geriatric patients attending a long-term care Spanish hospital. J Med Microbiol.

[40] Cavalcante FS, Saintive S, Carvalho Ferreira D, Rocha Silva AB, Guimarães LC, Braga BS, Dios Abad ED, Ribeiro M, Netto Dos Santos KR (2021). Methicillin-resistant Staphylococcus aureus from infected skin lesions present several virulence genes and are associated with the CC30 in Brazilian children with atopic dermatitis. Virulence.

[41] Li T, Yu X, Xie J, Xu Y, Shang Y, Liu Y, Huang X, Qin Z, Parsons C, Hu L (2013). Carriage of virulence factors and molecular characteristics of Staphylococcus aureus isolates associated with bloodstream, and skin and soft tissue infections in children. Epidemiol Infect..

[42] El-baz R, Rizk DE, Barwa R, Hassan R (2016). Virulence factors profile of Staphylococcus aureus isolated from different clinical sources. J Microbiol.

[43] Mirzaee M, Najar Peerayeh S, Ghasemian A-M (2014). Detection of icaABCD genes and biofilm formation in clinical isolates of methicillin resistant *Staphylococcus aureus*. Iran J Pathol.

[44] Chen X, Wu Z, Zhou Y, Zhu J, Li K, Shao H, Wei L (2017). Molecular and virulence characteristics of methicillin-resistant *Staphylococcus aureus* in burn patients. Front Lab Med.

[45] Eftekhar F, Rezaee R, Azad M, Azimi H, Goudarzi H, Goudarzi M (2017). Distribution of adhesion and toxin genes in *staphylococcus aureus* strains recovered from hospitalized patients admitted to the ICU. Arch Pediatr Infect Dis.

[46] Sedaghat H, Narimani T, Esfahani BN, Mobasherizadeh S, Havaei SA (2021). Comparison of the prevalence of microbial surface components recognizing adhesive matrix molecules (MSCRAMMs) among *Staphylococcus aureus* isolates in a burn unit with non-burning units. Iran J Public Health.

[47] Sabouni F, Mahmoudi S, Bahador A, Pourakbari B, Sadeghi RH, Ashtiani MTH, Nikmanesh B, Mamishi S (2014). Virulence factors of *Staphylococcus aureus* isolates in an Iranian referral children's hospital. Osong Public Health Res Perspect.

[48] Li X, Fang F, Zhao J, Lou N, Li C, Huang T, Li Y (2018). Molecular characteristics and virulence gene profiles of *Staphylococcus aureus* causing bloodstream infection. Braz J Infect Dis.

[49] Johnson WM, Tyler S, Ewan E, Ashton F, Pollard D, Rozee K (1991). Detection of genes for enterotoxins, exfoliative toxins, and toxic shock syndrome toxin 1 in *Staphylococcus aureus* by the polymerase chain reaction. J Clin Microbiol.

[50] Peacock SJ, Moore CE, Justice A, Kantzanou M, Story L, Mackie K, O'Neill G, Day NP (2002). Virulent combinations of adhesin and toxin genes in natural populations of *Staphylococcus aureus*. Infect Immun.

[51] Nashev D, Toshkova K, Salasia SIO, Hassan AA, Lämmler C, Zschöck M (2004). Distribution of virulence genes of *Staphylococcus aureus* isolated from stable nasal carriers. FEMS Microbiol Lett.

