# Supplementary material for: Virulence genes distributed among Staphylococcus aureus causing wound infections and their correlation to antibiotic resistance
Source: BMC Infect Dis. 2022 Jul 28;22:652. doi: 10.1186/s12879-022-07624-8 (PMC9547454; doi:10.1186/s12879-022-07624-8)

Figure S1. Detection of amplification product of *hla* gene by PCR; lane 1: negative control, lane 2: positive control and lanes 3 to 10: positive PCR products (209bp)

Figure S2. Detection of amplification product of *sea* gene by PCR; lane 1: positive control, lane 2: negative control and lanes 3 to 11: positive PCR products (120bp)

Figure S3. Detection of amplification product of *icaA* gene by PCR; lane 1: positive control, lane 2: negative control and lanes 3 to 10: positive PCR products (770bp)

Figure S4. Detection of amplification product of *fnbA* gene by PCR; lanes 1 to 7: positive PCR products (1279bp), lane 8: positive control and lane 9: negative control





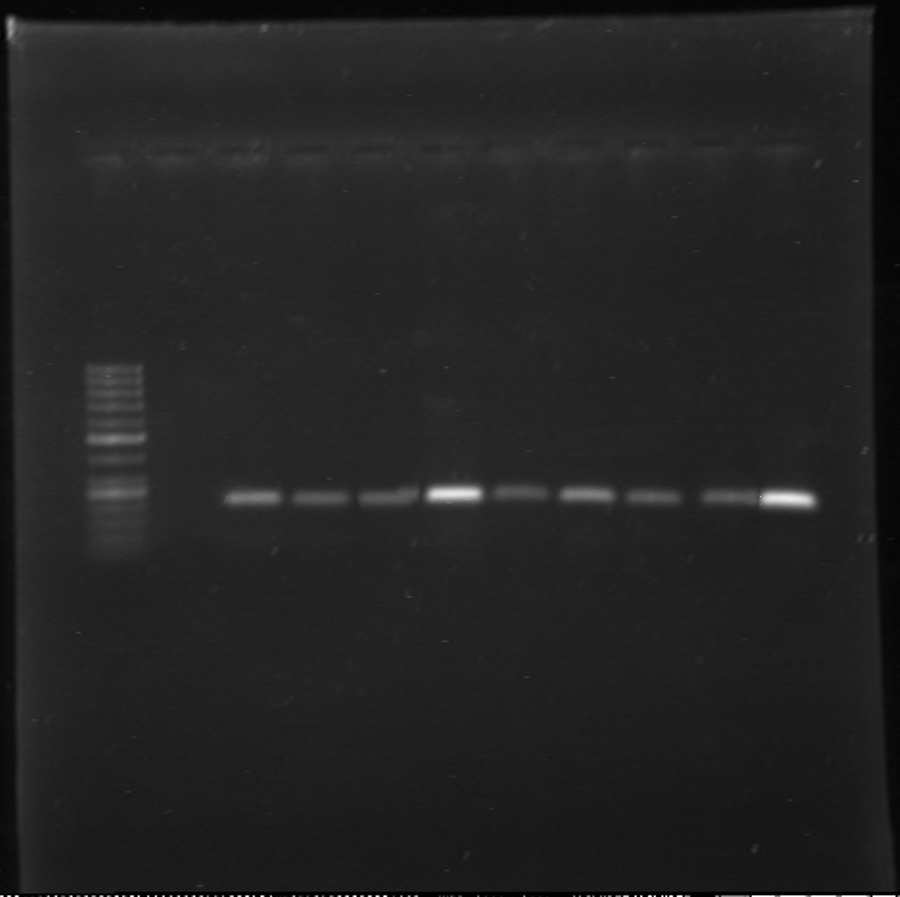


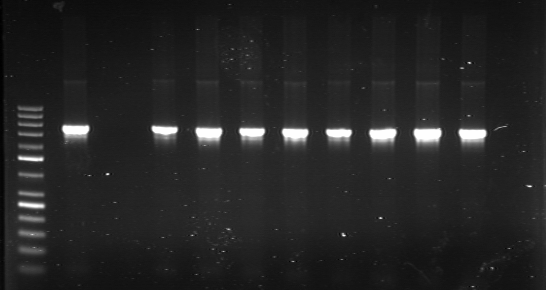


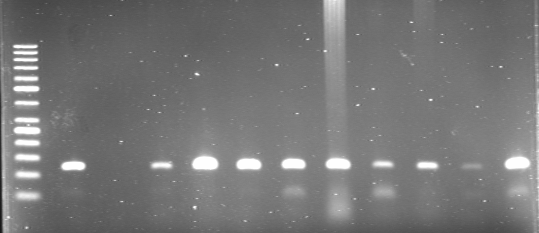

Supplement: Supplementary file 1 — Additional file 1: Figure S1. Detection of amplification product of hla gene by PCR; lane 1: negative control, lane 2: positive control and lanes 3 to 10: positive PCR products (209bp). Figure S2. Detection of amplification product of sea gene by PCR; lane 1: positive control, lane 2: negative control and lanes 3 to 11: positive PCR products (120bp). Figure S3. Detection of amplification product of icaA gene by PCR; lane 1: positive control, lane 2: negative control and lanes 3 to 10: positive PCR products (770bp). Figure S4. Detection of amplification product of fnbA gene by PCR; lanes 1 to 7: positive PCR products (1279bp), lane 8: positive control and lane 9: negative control [file 12879_2022_7624_MOESM1_ESM.docx]
